# hnRNP A1 interacts with the genomic and subgenomic RNA promoters of Sindbis virus and is required for the synthesis of G and SG RNA

**DOI:** 10.1186/1423-0127-17-59

**Published:** 2010-07-21

**Authors:** Hongxing Gui, Chi-Wei Lu, Sandra Adams, Victor Stollar, Mei-Ling Li

**Affiliations:** 1 Department of Physiology & Biophysics, Molecular Genetics, Microbiology & Immunology, UMDNJ-Robert Wood Johnson Medical School, Piscataway NJ 08854, USA; 2 Department of OB/GYN, UMDNJ-Robert Wood Johnson Medical School, Piscataway NJ 08854, USA; 3 Department of Biology & Molecular Biology, Montclair State University, Montclair NJ 07043, USA; 4 Department of Molecular Genetics, Microbiology & Immunology, UMDNJ-Robert Wood Johnson Medical School, Piscataway NJ 08854, USA; 5 The Cancer Institute of New Jersey, New Brunswick NJ 08903, USA

## Abstract

**Background:**

Sindbis virus (SV) is the prototype of alphaviruses which are a group of widely distributed human and animal pathogens. Heterogeneous nuclear ribonucleoprotein (hnRNP) A1 is an RNA-binding protein that shuttles between the nucleus and the cytoplasm. Our recent studies found that hnRNP A1 relocates from nucleus to cytoplasm in Sindbis virus (SV)-infected cells. hnRNP A1 binds to the 5' UTR of SV RNA and facilitates the viral RNA replication and translation.

**Methods:**

Making use of standard molecular techniques, virology methods and an in *vitro *system developed by our lab to assess the role of hnRNP A1 in SV positive strand RNA synthesis.

**Results:**

hnRNP A1 interacted with the genomic (G) and subgenomic (SG) RNA promoters. Knockdown of hnRNP A1 resulted in markedly decrease in the synthesis of G and SG RNA both in infected cells and *in vitro*.

**Conclusions:**

Our study provides the first direct evidence that hnRNP A1 actively participates in viral RNA replication and is required for the synthesis of G and SG RNA.

## Background

Alphaviruses are a group of widely distributed human and animal pathogens that includes almost 40 currently known members [1]. In natural conditions, most alphaviruses are transmitted by mosquitos that develop life-long chronic infections [2]. The presence of virus in the mosquito salivary glands mediates infection of vertebrates that serve as amplifying hosts. In vertebrates, alphaviruses develop acute infections characterized by high-titer viremia that is required for infection of mosquitoes during their blood meal. Ultimately, alphavirus infection of the vertebrate host is either cleared by the immune system or causes the death of an infected host.

Sindbis virus (SV) is the prototype virus of the family *Togaviridae*, genus *alphavirus *[1,3,4]. Sindbis virus has a non-segmented single-strand RNA genome of positive polarity, i.e. the genome can function as a messenger RNA. The genome is 11,703 nts in length, has a 5' type 0 cap and is polyadenylated at its 3' end.

In the infected cells, SV express only four nonstructural proteins, namely nsP1, nsP2, nsP3 and nsP4, which form, together with host factors, the enzyme complex responsible for the synthesis of virus-specific RNAs [5]. The nsP1 protein was found to have methyltransferase and guanylyltransferase activity, which are required for capping the newly synthesized, positive-strand viral RNAs [6-8]. It is palmitoylated and appears to be membrane-bound [9]. It also plays a role in the regulation of viral (-) strand RNA synthesis [10], and in the membrane attachment of the replication complex [11]. nsP2 was shown to be an RNA triphosphatase [12], RNA helicase [13] and a papain-like protease [14], and is responsible for the processing the P123 and P1234 polyproteins into the individual nsPs [3]. In addition, nsP2 is present not only in the replication complex, but was also found distributed in the cytoplasm and nuclei of infected cells [15,16]. nsP3 is the only nsP which is phosphorylated but its function is not yet clearly defined. The mutations in this protein can affect RNA replication, and, based on the structure of the replication complexes, this protein appears to have a structural function in the replication complex [17]. nsP4 is an RNA-dependent RNA polymerase (RDRP) [18] and possesses terminal adenylyltransferase activity [19].

Upon entry into the cell, the genome is translated into one of two polyproteins, P123 or P1234. These polyproteins are later processed in several steps by a viral protease into the four nonstructural (ns) proteins, nsP1, nsP2, nsP3, and nsP4. Initially, a complex of P123 (uncleaved nsP1, nsP2, and nsp3), and nsP4 form an RNA synthesizing complex that uses the incoming viral RNA as a template to synthesize a (-) strand RNA equivalent in length to the viral genome [5,20]. Upon further processing of the ns polyproteins, an RNA-synthesizing complex, now consisting of nsP1, nsP2, nsP3, and nsP4 is formed which uses the (-) strand RNA as a template to synthesize two (+) strand RNAs, a genomic (G) RNA, and a subgenomic (SG) RNA which has the same sequence as the terminal 3' one third of the viral genome. The SG RNA serves as the message for the three structural proteins, the capsid or C protein, and the two envelope proteins, E1 and E2. Because of stop codons after the nsP4 coding region, only the four ns proteins are translated from the G RNA.

In addition to virus-encoded factors, many steps in virus infections involve host factors. Such virus-host interactions are crucial determinants of virus host range, replication, and pathology, offer insights into viral and cellular function, and provide antiviral targets. Thus identifying such interactions and the associated host factors is a major frontier in virology [21,22]. Recently, using a human-genome-wide RNAi screen Krishnan et al. [23] have identified 305 host proteins that affect West Nile virus infection. Functional clustering of these genes revealed a complex dependence of this virus on a wide variety of host molecules and cellular pathways in viral infection. While the viral components of the SV replicase/transcriptase are known and to some extent characterized, the cellular proteins involved in viral RNA synthesis remain poorly understood. Moreover, while there is also strong evidence that host factors play a significant role in the activity of the replicase/transcriptase [24,25], the cellular proteins associated with the alphavirus nsPs and RNA remain to be characterized. The first reported interaction between a host protein and viral RNA was that between the mosquito La autoantigen and the 3' end of the SV negative strand RNA [26]. Recent work by Burnham et al. [27] has demonstrated that the host protein hnRNP K interacts with nsP2 and SG RNA in SV-infected cells. Several reports [17,28,29] have identified other cellular proteins associated with the SV replicase/transcriptase. These include cytoskeleton proteins, ribosomal subunits, chaperones, and hnRNPs. However, the roles of these cellular proteins in SV replication remain unclear. hnRNP A1 was not identified in these studies.

Members of the hnRNP family (including hnRNP A1) are involved in IRES-dependent translation of cellular and viral mRNA [30-32]. Moreover, hnRNPs are involved in several RNA metabolic processes, such as pre-mRNA splicing and trafficking [33,34] and telomerase regulation [35]. There is strong evidence that hnRNP A1 plays a critical role in telomere biogenesis [36]. Interestingly, hnRNP A1 has been shown to be involved in the replication of many viruses including mouse hepatitis virus [MHV], hepatitis C virus [HCV], dengue virus and human papillomavirus 16 [HPV 16], human cytomegalovirus, and vesicular stomatitis virus [VSV] [37-43].

Our recent studies reported that hnRNP A1 plays an essential role in the replication of SV [44]. hnRNP A1 interacted with the SV 5'UTR and facilitated the translation of viral RNA. Knockdown of hnRNP A1 reduced SV RNA synthesis and the yield of progeny virus. In the present study we extended the experiments on the role of hnRNP A1 in SV positive strand RNA synthesis. Our results suggest that hnRNP A1 associated with the genomic (G) and subgenomic (SG) RNA promoters and enhances the synthesis of G and SG RNA.

## Methods

### Cells and virus

BSC40 cells were cultured at 37°C in Eagle's minimum essential medium supplemented with 10% fetal calf serum (FCS) (Mediatech). CEF (chick embryo fibroblast) cells were grown in Dulbecco's modified Eagle medium (DMEM) supplemented with 10% FCS. SV was propagated in CEF cells. Cells were infected with SV at specified multiplicity of infection (moi) for 1 h at 34°C, washed away unbound inoculum, and refed with fresh medium. Media from infected cultures were harvested at the indicated times, and titers of SV were measured by plaque formation on CEF cells. Recombinant vaccinia viruses encoding Sindbis virus P123, nsP4, or T7 polymerase (VTF7-3), were kindly provided by Drs. Charles M. Rice and Richard Hardy and were propagated in BSC40 cells as described previously [45]. The Sindbis virus P123 contained the N614D change in nsP2 that results in more rapid processing of P123 than is observed with the wt P123[20,46]. The various nsPs were expressed from a T7 promoter.

### Preparation of labeled RNA probes and binding assay

24-mer and 45-mer oligoribonucleotides representing the negative strand sequence corresponding to nt 7579 to 7602 and nt 1-45 of the SV genome were synthesized by Dharmacon Research Inc. As already noted, these are the minimal sequences that have SG and G promoter activity respectively [47,48]. Oligoribonucleotides were labeled at their 5' ends using polynucleotide kinase and [γ-^32^P] ATP.

An EMSA was carried out to determine the interaction between the promoters and hnRNP A1 as described previously [47]. Briefly, 2 μg of hnRNP A1 was incubated for 30 min at 25°C with one of the ^32 ^P-labeled RNA probes (1×10^4 ^c.p.m). The reaction was carried out in binding buffer (10 mM Hepes, pH 7.5/150 mM KCl/0.5 mM EGTA/2 mM MgCl_2_/1 mM DTT/1 unit RNasin/10% glycerol) and the final volume of the reaction mixture was 10 μl. Binding of hnRNP A1 to the G and SG promoters was recognized by a slower migration of the labeled RNA probes.

### Northern blot analysis of G and SG RNA

BSC40 cells were infected with SV at an moi of 40 pfu/cell and at 8 h post infection total RNA was extracted using RNeasy mini kit (Qiagen). Five μg of total RNA was denatured with formaldehyde, and electrophoresed through a 1.5% agarose gel. The RNAs were transferred to a nylon membrane and subjected to northern blotting. The negative-strand probe, SV7772(-), which contains the negative-strand sequence from nt 7772 to 7754 were end-labeled with ^32 ^P-γ-ATP. After hybridization, the nylon membrane was washed twice with 2 × SSC and 0.1% SDS, exposed to a storage phosphor screen and scanned on a Molecular Dynamics Typhoon Phosphorimager.

### Expression and preparation of recombinant Sindbis virus nonstructural proteins

BSC40 monolayers in T75 tissue culture flasks were infected with three different recombinant vaccinia virus vectors; these encode the SV nonstructural polyprotein P123 (the polyprotein precursor of nsP1, nsP2, and nsP3), SV nsP4, and T7 RNA polymerase, each at an moi of one pfu/cell. Twenty to twenty four hours after infection, the P15 fraction was prepared according to Lemm et al. [45]. The P15 pellet isolated from one T75 flask was resuspended in 50 μl of storage buffer (10 mM Tris-Cl [pH 7.8], 10 mM NaCl, 15% glycerol) and used as the source of the Sindbis virus replicase/transcriptase (R/T) complex.

### *In vitro *synthesis of SV RNA

The reaction mixture for the *in vitro *synthesis of SV RNA (25 μl) contained 5 μl of 5 × reaction buffer (200 mM Tris-HCl, pH 7.9, 30 mM MgCl_2_, and 50 mM NaCl), 10 mM dithithreitol, 40 units of RNase inhibitor (Promega), 2 μg negative-strand RNA which serves as promoter/template (P/T), and 12.2 μl (5.4 μg of protein/μl) of P15 extract prepared from BSC40 cells infected with recombinant vaccinia viruses expressing the T7 RNA polymerase, the SV polyprotein, P123, and the SV nsP4. Standard reaction mixtures contained 3 mM ATP, 2 mM UTP, 2 mM CTP, and 0.5 mM GTP. [^32^P]α-GTP (800 Ci/mM, 10 μCi/μl) was included to label the transcripts. Incubation was at 37°C for 1 hour. RNA was extracted using phenol-chloroform and electrophoresed on a 1% denaturing agarose gel containing 2.2 M formaldehyde. The gel was transferred to a Genescreen Plus Hybridization Transfer Membrane (Perkin Elmer); the membrane was then exposed to a storage phosphor screen and scanned as described previously [49]. The negative-strand P/T for making both G and SG RNA was as described in Li et al. [48].

### Expression and purification of recombinant hnRNP A1 protein

hnRNP A1 cDNA derived from SF268 cellular mRNA was cloned into pET30a and expressed as described [44]. The recombinant hnRNP A1 protein was purified using a HisTrap kit (GE). Protein purity was determined by electrophoresis on 12% SDS-PAGE, and concentration was determined using the Bio-Rad protein assay.

### siRNA knockdown

BSC40 cells were maintained in antibiotic-free media. 100 nmol of siRNA targeting hnRNP A1 (ON-TARGETplus SMARTpool L-008221-00-0005, Dharmacon) was transfected along with 3 μl FuGENE HD transfection reagent (Roche) in 0.4 ml MEM supplemented with 10% FCS following manufacturer's directions. Three days after transfection cells were co-infected with recombinant vaccinia viruses expressing T7, SV-nsP123 and SV-nsP4 at an moi of one pfu/cell. P15 fraction was prepared as described [47]. An aliquot of cell lysate was subject to Western blot for analysis of protein expression.

### Western blotting

Expression of hnRNP A1 was examined by Western blotting using anti-hnRNP A1 antibody (Abcam). Briefly, cells were lysed in sample buffer and proteins were fractionated by SDS-PAGE in 12% polyacrylamide gels and transferred to PVDF membranes by wet transfer. Membranes were blocked with PBS containing 5% low-fat dry milk. Anti-hnRNP A1 mouse antibody was then added, and the membranes were washed with PBS containing 0.2% Tween 20. Goat anti-mouse horseradish peroxidase-conjugated antibody (BioRad) and the ECL kit (Pierce) were used to detect bound antibodies.

## Results

### hnRNP A1 interacts with the G and SG promoters of Sindbis virus

**Figure 1:**
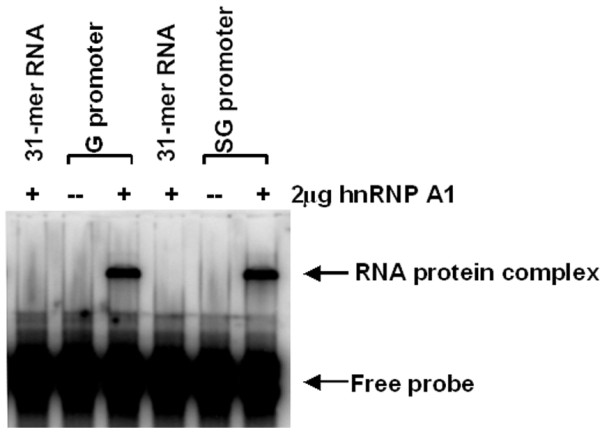
**Binding of hnRNP A1 to the G and SG promoters**. EMSA was performed as described under Material and Methods. The G and SG promoters were end-labeled with ^32^PO_4 _(1 × 10^4 ^cpm per reaction) and incubated at 37°C for 30 min either alone or with 2 μg of hnRNP A1. A non-specific 31-mer RNA oligo was end-labeled with ^32^PO_4 _and reacted with 2 μg of hnRNP A1

Our previous study demonstrated that knockdown of hnRNP A1 in infected cells reduces the viral RNA synthesis [44]. To further dissect the role of hnRNP A1 in viral RNA synthesis, we tested if hnRNP A1 is involved in the positive strand RNA synthesis by interacting with the promoters. To learn whether hnRNP A1 interacts with the G and SG promoters, we tested the binding of hnRNP A 1 to the SV G and SG promoters. An EMSA was carried out using hnRNP A1 and the labeled G and SG promoters of Sindbis virus. As shown in Fig. 1, hnRNP A1 did not bind to a non-specific 31-mer RNA oligo but did bind to the SV G and SG promoters slowing their electrophoretic mobility. These results indicate that the interaction between the G and SG promoters and hnRNP A1 is specific.

### hnRNP A1 is required for the synthesis of G and SG RNA in infected cells

**Figure 2:**
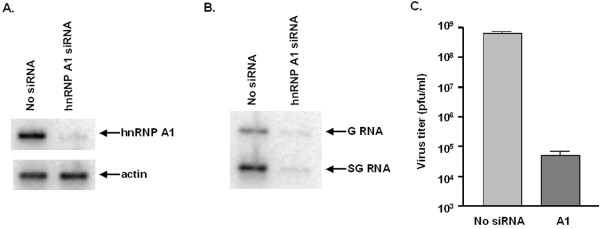
**Effect of hnRNP A1 on the synthesis of G and SG RNA in infected cells**. **(A) **Western blot to examine the expression of hnRNP A1. hnRNP A1 was depleted by siRNA knockdown technique as described under Materials and Methods. Cell lysates were prepared 3 days after transfection of siRNA. Western blot was carried out using antibody to hnRNP A1. Expression of actin was examined by Western blot as a control. **(B) **Northern blot to detect G and SG RNA in infected cells. Cells were transfected with no siRNA or siRNA targeting hnRNP A1 for 3 days. Cells were infected with SV at an moi of 40 pfu/cell for 8 h. Total RNA were extracted and electrophoresed through an agarose gel, transferred to a nylon membrane, and characterized by Northern blot analysis, using ^32^P-labeled negative-strand probe SV7772(-). **(C) **Replication of SV in hnRNP A1-depleted cells. Cells treated or untreated with siRNA targeting hnRNP A1 were infected with SV at an moi. of 40 pfu/cell and incubated at 34°C. Medium was harvested 8 h p.i. and assayed for infectious virus by plaque formation on CEF cells. Mean values and standard errors from duplicate samples are shown.

While we have good evidence that hnRNP A1 binds to the G and SG promoters, the consequences of this interaction for viral RNA synthesis are not known. Our recent studies showed that hnRNP A1 re-localizes from nucleus to cytoplasm of SV-infected cells. Knockdown of hnRNP A1 inhibits the virus replication [44]. These findings led us to hypothesize that the binding of hnRNP A1 to the G and SG promoters is involved in the regulation of G and SG RNA synthesis during the virus replication cycle. To assess the role of hnRNP A1 in viral positive strand RNA synthesis, we knocked down the expression of hnRNP A1 in infected cells and examined the synthesis of G and SG RNA by Northern blot. hnRNP A1 was depleted by siRNA targeting hnRNP A1 in BSC40 cells as described previously [44]. Knockdown efficiency was examined by Western blot using anti-hnRNP A1 antibody (Abcam). As shown in Fig. 2A, expression of hnRNP A1 was diminished in cells transfected with siRNA targeting hnRNP A1. Cells were then infected with SV at an moi of 40 pfu/cell for 8 h. Total RNA was extracted and subject to Northern blot as described in Materials & Methods. As shown in Fig. 2B, the synthesis of G and SG RNA was dramatically inhibited when hnRNP A1 was depleted indicating hnRNP A1 is essential for the positive strand RNA synthesis. The medium was collected and plaque assay was carried out to determine the virus titer, as shown in Fig. 2C, the titer dropped about 4 log in hnRNP A1 knockdown cells.

### hnRNP A1 enhances the synthesis of G and SG RNA *in vitro*

We made use of an *in vitro *system for the synthesis of G and SG RNA developed by our laboratory [48,49] to examine the role of hnRNP A1 in the synthesis of G and SG RNA. Synthesis of the G and SG RNA transcript was followed by including ^32^P-α-labeled GTP in the reaction mixture, along with the four unlabeled NTPs, the P/T, and the P15 fraction as described in Materials & Methods.

**Figure 3:**
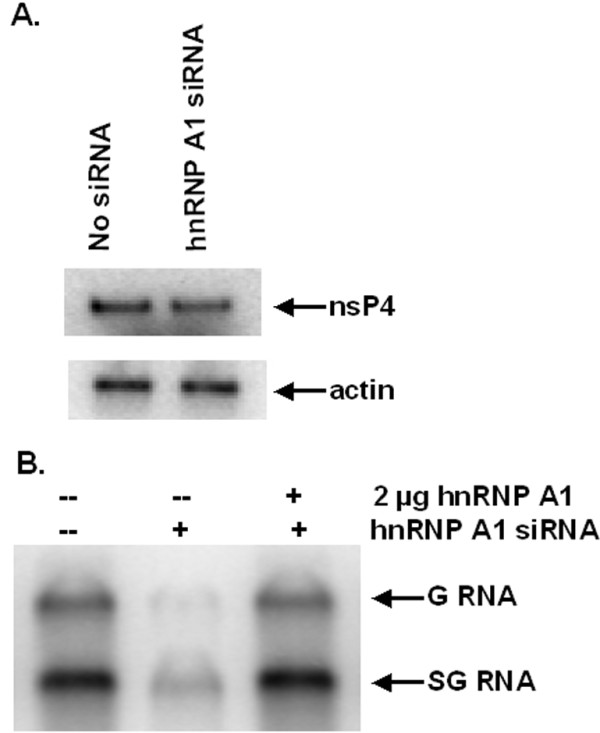
**Effect of hnRNP A1 on the synthesis of G and SG RNA *in vitro***. **(A) **Western blot to examine the expression of nsP4. BSC40 cells were transfected with no siRNA or siRNA targeting hnRNP A1 for 3 days as described under Materials and Methods. Cells were co-infected with recombinant vaccinia viruses expressing T7 RNA polymerase, SV nsP123 and SV nsP4 at an moi of one for 24 h. Cell lysates were prepared and subjected to 12% SDS-PAGE electrophoresis. Western blot was carried out using antibody to nsP4. Expression of actin was examined by Western blot as a control. **(B) ***in vitro *G and SG RNA synthesis. hnRNP A1 was depleted from BSC40 cells as described above. Cells were co-infected with recombinant vaccinia viruses expressing T7 RNA polymerase, SV nsP123 and SV nsP4 and P15 fractions were prepared as described under Materials and Methods. P/T were prepared and standard reaction conditions were used as described under Materials and Methods.

The level of endogenous hnRNP A1 in BSC40 cells was knocked down by siRNA targeting of hnRNP A1 as described in Materials & Methods. The knockdown efficiency was confirmed by Western blot using anti-hnRNP A1 antibody (data not shown). The hnRNP A1-depleted BSC40 cells were then co-infected with recombinant vaccinia viruses expressing T7 polymerase, SV P123 and SV nsP4 and the p15 were prepared as described [47]. As shown in Fig. 3A the expression of nsP4 in hnRNP A1-depleted cells was as good as in wild type cells indicating that knockdown of hnRNP A1 did not affect the expression of the SV nonstructural proteins. To see if knockdown of hnRNP A1 affects G and SG RNA synthesis *in vitro*, the reaction was set up as described [48] using either normal or hnRNP A1-depleted replicase/transcriptase (P15 fraction) and the P/T which contains both 5' and 3' termini and the SG promoter region. As shown in Fig. 3B depletion of hnRNP A1 in BSC40 cells resulted in decreased synthesis of both G and SG RNA. Addition of purified hnRNP A1 back to the hnRNP A1-depleted replicase/transcriptase was able to restore the RNA synthesis. The results indicate that hnRNP A1 is required for G and SG RNA synthesis.

## Discussion

Host factors together with viral proteins regulate virus RNA replication. The finding that mutations in the viral genome sometimes have different effects in vertebrate cells and mosquito cells has suggested that cellular factors play a role in viral RNA synthesis [7]. Recent studies from several groups have identified cellular proteins associated with the SV RNA replication complex by isolating nonstructural protein-containing complexes from infected cells [17,27-29]. However, the roles that most of these proteins play in the replication of SV remain to be elucidated. Our recent study found that after SV infection hnRNP A1 relocalized from nucleus to cytoplasm, where the SV replication occurs. hnRNP A1 was able to bind to the capped 5'UTR of SV RNA and knockdown of hnRNP A1 diminished the SV viral RNA and protein synthesis, cap-dependent translation and viral replication. We therefore conclude that hnRNP A1 promotes SV replication via binding to the 5'UTR and facilitates possibly both viral RNA synthesis and translation [44].

How does hnRNP A1 contribute to the replication of SV? As hnRNP A1 is an RNA-binding protein, we speculated that it might facilitate viral RNA synthesis via association with viral RNA. In our previous study we found that hnRNP A1 interacts with the 5'UTR of SV RNA and promotes the synthesis of negative strand RNA [[44], data not shown]. In the present study we demonstrated that hnRNP A1 specifically interacts with both the G and SG promoters and enhances the synthesis of G and SG RNA as knockdown of hnRNP A1 rendered dramatically decrease in the synthesis of these two RNA species. Taken together we demonstrate that hnRNP A1 is required for the synthesis of both positive and negative strand RNA.

G and SG promoters are different in sequences. Our previous study showed that nsP4, the RDRP, has distinct sites for the binding of G and SG promoters [47,48]. We therefore suggest that in a similar fashion, hnRNP A1 also has different sites for the recognition of these two promoters. In future work we intend to use the same procedures that were used to identify the G and SG promoter binding sites in nsP4 [47] to identify the binding sites for G and SG promoters in hnRNP A1.

How does hnRNP A1 contribute to the viral RNA replication? hnRNP A1 is predominantly a nuclear protein. During SV infection nearly all of hnRNP A1 relocalizes to cytoplasm in association with viral RNA. Precisely what interactions lead to the recruitment of hnRNP A1 to the viral RNA replication complex is unknown. As hnRNP A1 is an RNA-binding protein, it may direct the viral RNA replication complex containing the viral RDRP, other viral and cellular factors to the sites where replication of viral RNA is initiated. Moreover, considering the reported physiological roles of hnRNP A1, we speculate that hnRNP A1 may interact with viral nonstructural proteins which comprise the viral RNA replication complex and facilitates the viral RNA replication. Further investigation is going on to address how hnRNP A1 contributes to viral RNA replication.

To our knowledge, this is the first report to describe an RNA-binding protein (hnRNP A1) actively participating in SV RNA replication in molecular detail. These studies not only improve our understanding of the replication of SV, but also have the potential for use as the basis for developing antiviral agents that act by inhibiting the virus RNA replication.

## Conclusion

Our previous study and the present report demonstrate that hnRNP A1 is essential for the RNA replication of Sindbis virus. hnRNP A1 binds to the 5' UTR of SV RNA and facilitates the negative strand RNA synthesis. Moreover hnRNP A1 interacted with the genomic (G) and subgenomic (SG) RNA promoters and is required for the synthesis of G and SG RNA both in infected cells and *in vitro*. Our study provides the first direct evidence that hnRNP A1 actively participates in viral RNA replication and is required for the synthesis of G and SG RNA.

## Competing interests

The authors declare that they have no competing interests.

## Authors' contributions

HXG designed the experiments and carried out the in vitro RNA synthesis, virus titration and drafted the manuscript, CWL and SA carried out the Northern and Western blots, SA proof read the manuscript, VS and MLL designed and troubleshoot the experiments, and prepared the manuscript. All authors read and approved the final manuscript.
